# Quantity discrimination in newly hatched zebrafish suggests hardwired numerical abilities

**DOI:** 10.1038/s42003-023-04595-7

**Published:** 2023-03-23

**Authors:** Tyrone Lucon-Xiccato, Elia Gatto, Camilla Maria Fontana, Angelo Bisazza

**Affiliations:** 1 Department of Life Sciences and Biotechnology, University of Ferrara, Ferrara, Italy; 2 Department of Chemical, Pharmaceutical and Agricultural Sciences, University of Ferrara, Ferrara, Italy; 3 Department of Biology, University of Padova, Padova, Italy; 4 Department of General Psychology, University of Padova, Padova, Italy; 5 Padova Neuroscience Center, University of Padova, Padova, Italy

**Keywords:** Animal behaviour, Evolution, Psychology

## Abstract

An intriguing hypothesis to explain the ubiquity of numerical abilities is that all vertebrates are born with hardwired neuronal networks for processing numbers. To date, only studies on human foetuses have clearly supported this hypothesis. Zebrafish hatch 48–72 h after fertilisation with an embryonic nervous system, providing a unique opportunity for investigating this hypothesis. Here, we demonstrated that zebrafish larvae exposed to vertical bars at birth acquired an attraction for bar stimuli and we developed a numerical discrimination task based on this preference. When tested with a series of discriminations of increasing difficulty (1vs.4, 1vs.3, 1vs.2, and 2vs.4 bars), zebrafish larvae reliably selected the greater numerosity. The preference was significant when stimuli were matched for surface area, luminance, density, and convex hull, thereby suggesting a true capacity to process numerical information. Converging results from two phylogenetically distant species suggests that numerical abilities might be a hallmark feature of vertebrates’ brains.

## Abstract (web summary)

Newly-hatched zebrafish larvae show true capacity to process numerical information, suggesting that all vertebrates might have hardwired numerical abilities.

## Introduction

Mathematical competence has played a crucial role in advancing human civilization and technological culture. However, numerical abilities occur in different forms and with various degrees of sophistication across all vertebrate taxa, including cartilaginous fishes^1^, teleost fishes^2^, amphibians^3^, reptiles^4^, birds^5^, and mammals^6,7^. A popular but controversial hypothesis to explain the ubiquity of numerical abilities is that the vertebrate brain contains specific circuits specialised to encode and process quantitative information^8–11^, as observed for other cognitive functions (e.g., basic learning and memory^12–14^; object recognition^15,16^).

If circuits hardwired to process quantitative information are fundamental components of the vertebrate brain, as several authors have hypothesised^8–11^, we expect numerical abilities to appear already operational during early brain development. Unfortunately, early development of numerical abilities has been typically investigated in precocial species (guppies^17^ and chickens^18^). Because the young of precocial species are born or hatched after a long developmental period, their brain and behavioural repertoire closely match that of adults^19–22^. Therefore, this well-developed brain might allow newborns to cope with numerical tasks with compensatory mechanisms^23,24^. To study the early appearance of cognitive abilities, it is possible to focus on vertebrate species that have evolved developmental modes as opposed to precociality^25–27^. In humans, the only altricial species whose cognitive development has been studied in detail, behavioural and imaging evidence suggests numerical processing in newborns^28,29^ and foetuses^30^. These findings align with the idea of the early development of numerical abilities but are difficult to generalise to other species. The cortex’s exceptional expansion and the computational potential of the human brain might determine enhanced, generalised cognitive functionality even during early development^26,31^.

Therefore, at the current research stage, it is critical to investigate non-human species that have short embryonic periods and show an immature nervous system at birth to test the hypothesis of hardwired vertebrate numerical abilities. For this aim, we exploited the zebrafish, *Danio rerio*, a teleost fish displaying a developmental mode similar to extreme altriciality. The zebrafish larvae hatch 48-72 h post fertilization (hpf) and tend to be inactive, lying on one side on the benthos, for a few days. Free swimming is observed 120 hpf, after the inflation of the bladder, and feeding is observed 140 hpf, when the digestive tract’s development is completed. The visual system starts to develop early (response to light: 72 hpf; tracking eye movements: 80 hpf), permitting the exploitation of available numerical tests that typically rely on visual stimuli. Therefore, zebrafish’s particular developmental biology is a key tool for testing the hypothesis of hardwired circuities for numerical abilities and for understanding the development of cognitive abilities in general.

To examine zebrafish larvae’s numerical abilities as early as possible, we avoided the lengthy associative learning paradigms generally used in the field^4,32^ and we conceived of a rapid free-choice test for relative quantity discrimination^17,28^. Considering the limited behavioural repertoire zebrafish larvae display, we focused on the simple choice between clusters of vertical bar stimuli that simulated habitats with different amounts of vegetation^3,33^. Attraction to environmental features is spontaneous in some species such as the goldfish^34^, but in others, it reflects the habitat in which the individual grows^35,36^. In zebrafish, both choice mechanisms could potentially apply, given that in its natural range, this species occupies habitats both with and without vegetation^37,38^. Therefore, in our first experiment, we verified whether newly hatched zebrafish larvae display or can develop an attraction to clusters of vertical bars simulating vegetation. In the second part of the study, we used our free-choice procedure to investigate zebrafish larvae’s ability to discriminate between different numbers of bars. We additionally performed a series of control tests to assess the influence of non-numerical stimulus features that naturally covary with numbers^39^. If the hypothesis of innate numerical abilities in vertebrates holds true, we expect to observe the capacity to discriminate the stimuli based on numerical information in newly hatched zebrafish larvae.

## Results

### Zebrafish larvae acquire a preference for vertical bar stimuli

**Fig. 1:**
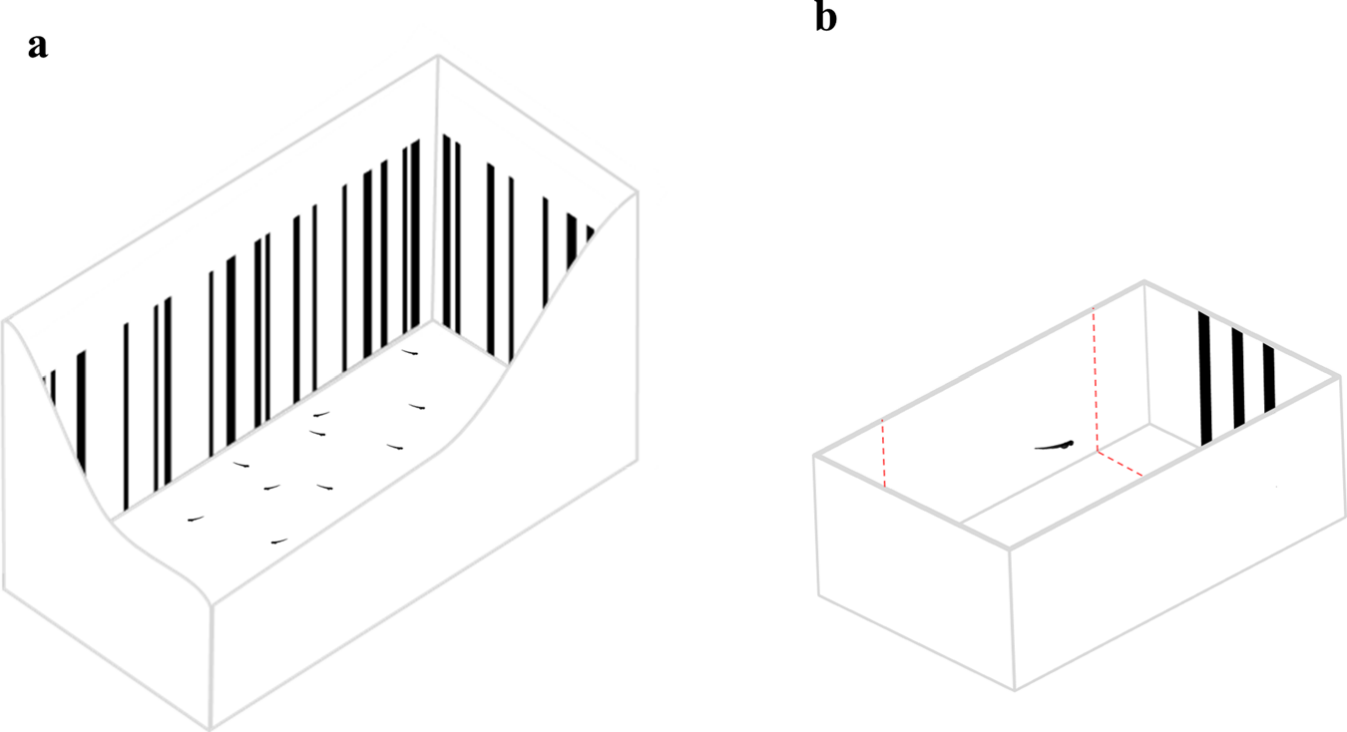
Paradigm developed to investigate zebrafish larvae’ numerical abilities. **a** Groups of larvae were reared in aquaria with walls with bars since hatching. **b** The preference for bar stimuli was tested with a dichotomous-choice test, recording the time spent in proximity of the stimuli.

**Fig. 2:**
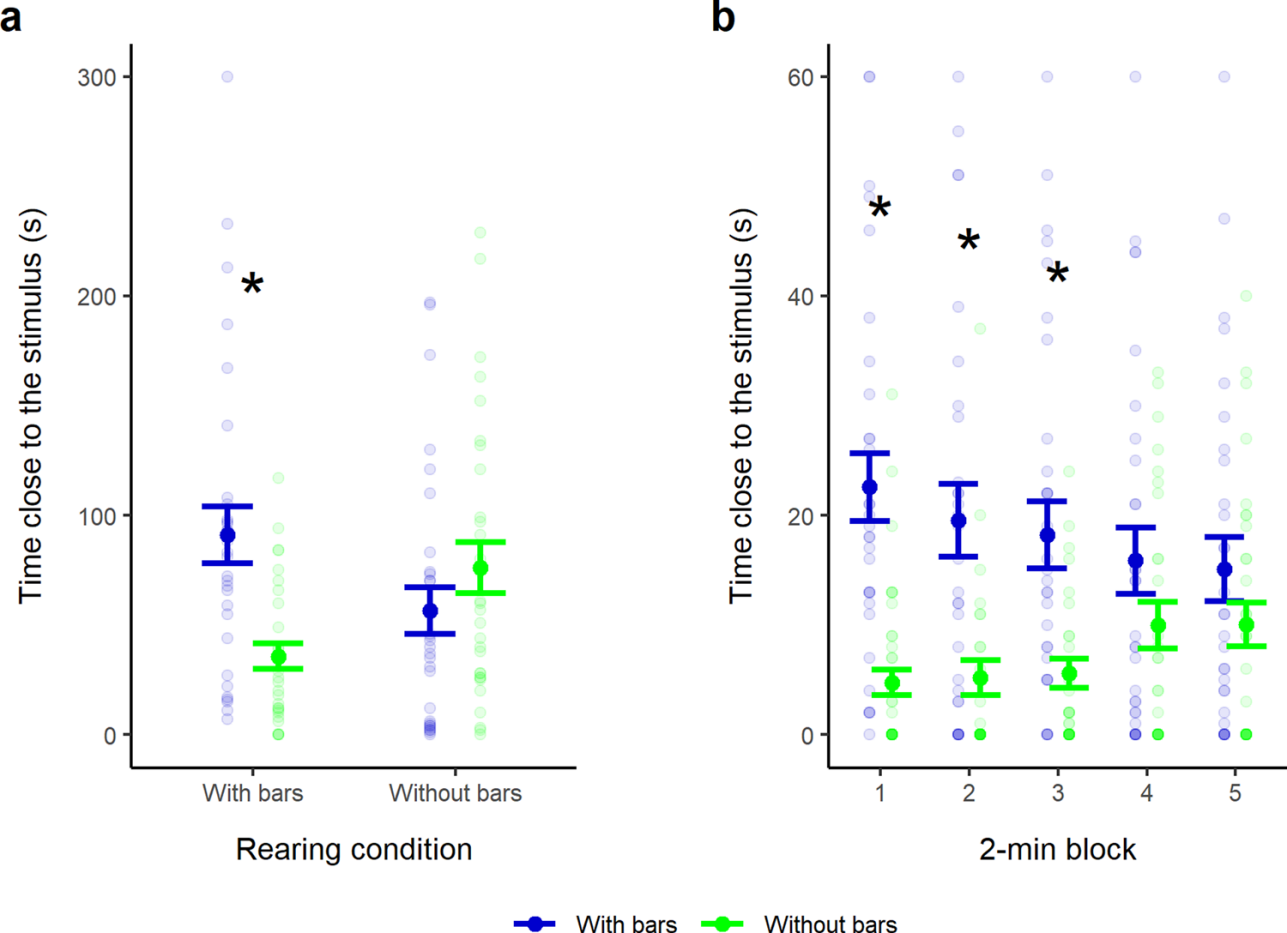
Preference for bar stimuli in a dichotomous choice test. **a** Preference for the choice sector with bar stimuli (blue) and for the choice sector without stimuli (green), divided according to the rearing aquaria of the subjects (N = 60 subjects). **b** Temporal variation in preference for vertical bars in larvae raised in the aquaria with bars (*N* = 60 subjects). Large dots and error bars represent means and standard errors, respectively. Small dots indicate individual data points. Asterisks represent a significant difference (*P* < 0.05) in the time spent in the two choice sectors.

We assessed attraction towards the bar stimuli in a dichotomous free-choice test using larvae raised in small aquaria either with black vertical bars on the walls or with uniform white walls (Fig. 1a, b). The larvae behaved differently according to the type of aquarium in which they were raised (linear mixed-effects model: *F*_1,116_ = 14.50, *P* < 0.001; Supplementary Table 1). In the testing apparatus, larvae raised in an environment with bars showed a significant preference for the choice sector with the bar stimuli (preference for the stimulus with bars: 66.11 ± 26.93%; paired-samples *t*-test: *t*_29_ = 3.38, 95% CI [1.44, 5.88], *P* = 0.002), whereas subjects raised in the aquaria without bars showed no preference between the two choice sectors (preference for the stimulus with bars: 41.16 ± 33.39%; *t*_29_ = −1.09, 95% CI [−4.17, 1.28], *P* = 0.286; Fig. 2a). This indicated that a preference for vertical bars can be induced by exposing larvae to an environment with this feature, in contrast to what is observed in other fish species in which similar preferences are innate^34^. The mechanism behind this plastic preference might relate to behavioural attraction towards familiar habitats but it might also involve physiological changes in the visual system’s sensitivity^40^. Critically, the induced preference could be used to study numerical discriminations.

A temporal analysis demonstrated that the preference shown by subjects raised in aquaria with bars was marked at the beginning of the test, but weakened after a few minutes (linear mixed-effects model: *F*_1,267_ = 11.04, *P* = 0.001; Fig. 2b; Supplementary Table 2). We found a significant preference for the sector with the bars from minute 1 to minute 6 of the test (block of minutes 1–2: *t*_29_ = 4.61, 95% CI [1.49, 3.87], *P* < 0.001, minutes 3–4: *t*_29_ = 3.91, 95% CI [1.12, 3.59], *P* < 0.001, and minutes 5–6: *t*_29_ = 4.00, 95% CI [1.16, 3.57], *P* < 0.001), but not between minute 7 and minute 10 (minutes 7–8: *t*_29_ = 1.09, 95% CI [−0.68, 2.24], *P* = 0.283; minutes 9–10: *t*_29_ = 1.04, 95% CI [−0.72, 2.21], *P* = 0.307). The preference did not significantly vary across the three intervals in which it was greater than chance (minutes 1–2, 3–4, and 5–6: *F*_1,147_ = 0.20, *P* = 0.652). The observed temporal trend suggested that the preference might be due to subject’s seeking refuge in a familiar habitat (i.e., with bars) when exposed to the novel environment of the testing apparatus. The preference attenuated over time likely because the larvae habituated to the novel environment and reduced the motivation to hide^41^. These results suggested using a 6-min testing time in the following experiments.

### Larval zebrafish discriminate between different numbers of bar stimuli

**Fig. 3:**
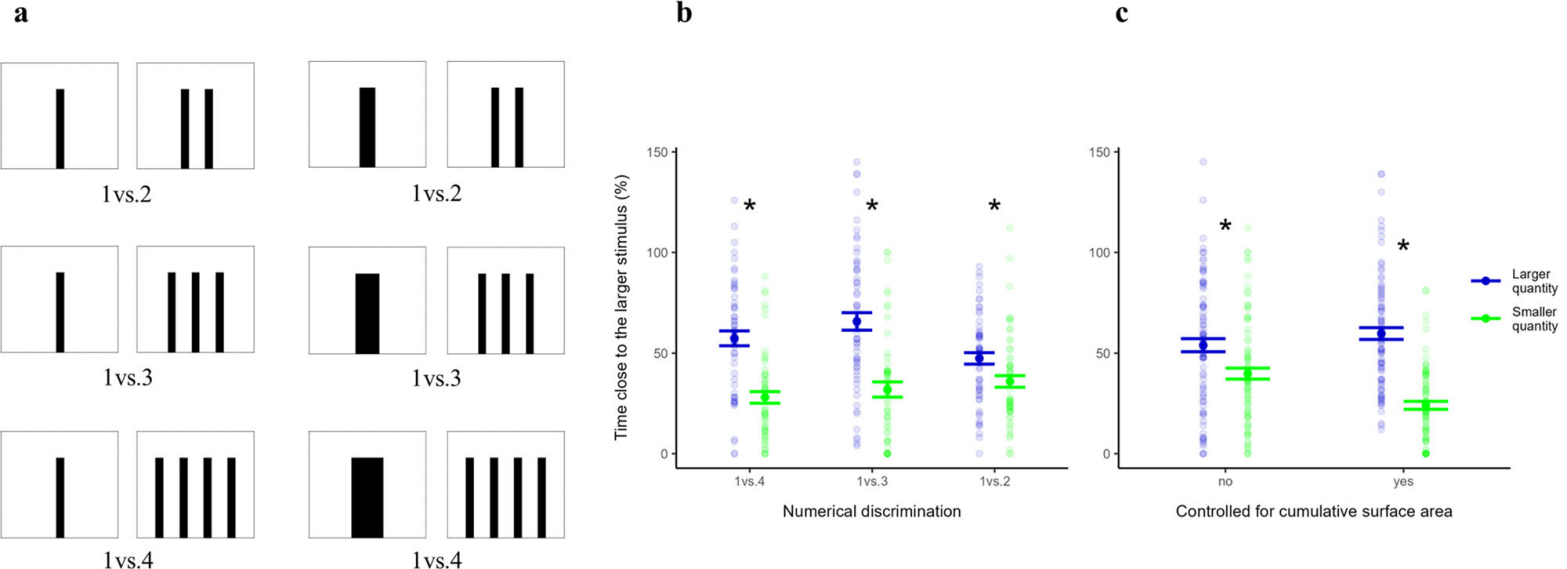
Discrimination of bar stimuli with different numerosity in zebrafish larvae. **a** Bar stimuli used in the first numerical discrimination experiment. **b** Preference for the choice sector with the larger numerosity and for the choice sector with the smaller numerosity, divided according to the numerical ratio of the stimuli (*N* = 180 subjects); large dots and error bars represent means and standard errors, respectively; small dots indicate individual data points; asterisks represent a significant difference (*P* < 0.05) in the time spent in the two choice sectors. **c** Preferen**c**e for the choice sector with the larger numerosity and for the choice sector with the smaller numerosity divided according to the stimuli’s control type (*N* = 180 subjects); large dots and error bars represent means and standard errors, respectively; small dots indicate individual data points; asterisks represent a significant difference (*P* < 0.05) in the time spent in the two sectors.

Results of the prior experiment demonstrated that larvae raised in the environment with bars, but not those raised in the white environment, displayed a preference for bar stimuli. We exploited this induced preference to assess numerical functions in newly hatched zebrafish. Naïve subjects raised in the environment with bars were presented with three discriminations of varying difficulty, 1vs.4, 1vs.3, and 1vs.2 bars, either with identical bars (i.e., bars that were of the same width) or bars that varied in width so as to equate cumulative surface area and the luminance between the two stimuli (Fig. 3a). We did this because humans and other vertebrates often use these latter cues as an alternative to solve quantitative tasks^42,43^.

Overall, zebrafish larvae showed a significant preference for the sector with the larger number of bars (preference for the larger quantity: 63.12 ± 24.39%; *F*_1,348_ = 85.18, *P* < 0.001; Supplementary Table 3); the time spent in the sector with the larger quantity (56.9 ± 29.4 s) was nearly twice that spent in the sector with the smaller quantity (32.0 ± 23.7 s). The model also revealed a significant interaction between stimulus (larger or smaller quantity) and ratio (*F*_2,348_ = 6.44, *P* = 0.002; Fig. 3). Analyses separated for each numerical ratio showed that the preference for the larger number of bars was always significant (1 vs. 4: preference for the larger quantity 66.53 ± 24.90%, *t*_59_ = 5.30, 95% CI [18.31, 40.49], *P* < 0.001; 1 vs. 3: 65.84 ± 26.16%, *t*_59_ = 4.76, 95% CI [19.67, 46.17], *P* < 0.001; 1 vs. 2: 56.98 ± 21.05%, *t*_59_ = 2.54, 95% CI [2.46, 20.47], *P* = 0.013; Fig. 3b). However, the preference weakened with increasing discrimination difficulty (Estimate −15.39 ± 6.62, *t*_348_ = 3.33, *P* = 0.021; Fig. 3b).

In addition, the overall analysis revealed a significant interaction between stimulus and control type (*F*_2,348_ = 15.97, *P* < 0.001; Fig. 3c). A separated analysis indicated that zebrafish larvae significantly preferred the larger number of bars in both the set of stimuli not controlled for the cumulative area and luminance (preference for the larger quantity: 56.15 ± 25.90%; *t*_59_ = 2.76, 95% CI [3.96, 24.31], *P* = 0.007) and in the set of stimuli controlled for these two variables (70.08 ± 20.67%; *t*_89_ = 8.37, 95% CI [27.24, 44.21], *P* < 0.001). Therefore, zebrafish larvae did not use the bars’ cumulative surface area and their luminance as a proxy for number.

The preference for the larger numerosity also emerged when considering the first block of minutes (time spent in the sector with the larger quantity: 21.1 ± 14.76 s; time spent in the sector with the smaller quantity: 9.03 ± 11.17 s: *F*_1,348_ = 84.15, *P* < 0.001; Supplementary Table 3). The analysis on the first block further revealed a significant interaction between stimulus and numerical ratio (*F*_2,348_ = 7.04, *P* = 0.001) and a significant interaction between stimulus and control type (*F*_2,348_ = 5.21, *P* = 0.023), in line with the main analysis.

### Density of items and the extension of the array do not affect numerical discrimination

**Fig. 4:**
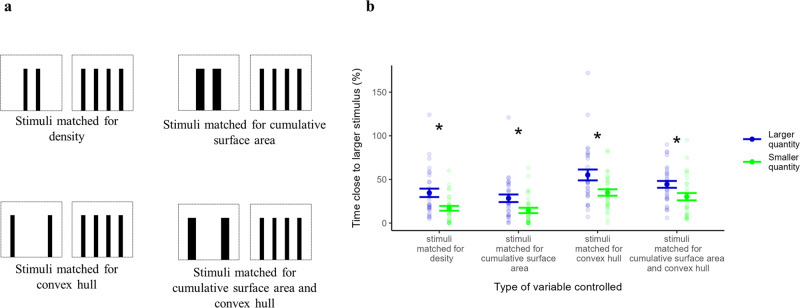
Discrimination of numerical stimuli controlled for continuous variables. **a** Stimuli used in the 2vs.4 bars discrimination performed to unravel the role of continuous variables in quantitative discrimination. **b** Preference for the choice sector with the larger (four) and the smaller (two) number of bars with respect to the four continuous variables controlled (*N* = 120 subjects). Large dots and error bars represent means and standard errors, respectively. Small dots indicate individual data points. Asterisks represent a significant difference (*P* < 0.05) in the time spent in the two sectors.

In a previous experiment, we could control for the effect of cumulative surface area, but vertebrates can also rely on other physical attributes of the stimulus, in particular, the density of items and the extension of the array (convex hull) to process numerosity^32,43,44^. Therefore, we tested naïve larvae in the most difficult ratio (0.5), giving the choice between two groups of bars, 2 vs. 4, allowing us to control the stimuli’s density and the convex hull (Fig. 4a). Overall, the larvae preferred the larger numerosity (time spent in the sector with the larger quantity: 40.63 ± 28.42 s; time spent in the sector with the smaller quantity: 24.15 ± 20.6 6 s; preference for the larger quantity: 63.23 ± 25.88%; *F*_1,232_ = 37.80, *P* < 0.001; Fig. 4b; Supplementary Table 4), supporting prior experiment’s conclusions. More important, the preference for the larger group of bars was significant even considering each control condition (stimuli matched for density: preference for the larger quantity: 65.63 ± 26.62%, *t*_29_ = 3.078 95% CI [0.66, 3.26], *P* = 0.005; stimuli matched for cumulative surface area and luminance: 68.95 ± 29.86%, *t*_29_ = 2.84, 95% CI [0.52, 3.19], *P* = 0.008; stimuli matched for convex hull: 57.95 ± 22.26%, *t*_29_ = 2.23, 95% CI [0.12, 2.89], *P* = 0.034; stimuli controlled for both cumulative surface area and convex hull: 60.40 ± 23.99%, *t*_29_ = 2.28, 95% C.I. [0.14, 2.55], *P* = 0.030). The same preference for the larger numerosity emerged from the first block of minutes (time spent in the sector with the larger quantity: 12.75 ± 11.83 s; time spent in the sector with the smaller quantity 6.53 ± 7.80 s; *F*_1,232_ = 30.32, *P* < 0.001; Supplementary Table 4). Therefore, none of the continuous variables was critical to determine the preference for the larger quantity of larvae, leaving the number as the only reliable information to explain subjects’ preference.

## Discussion

Although the list of known vertebrate species capable of solving numerical tasks is steadily growing, uncertainty remains on whether cognitive functions for numerical computation are a constitutive feature of vertebrates. We tried to shed light on this question by studying numerical abilities in recently hatched zebrafish larvae. We discovered that larval zebrafish acquired a preference for vertical bar stimuli after being habituated from hatching to a background with similar characteristics. When assayed in a dichotomous choice test between clusters of bars with different numerosity, these larvae selected the larger quantity.

A major challenge of studies on numerical cognition is to ascertain experimentally whether an animal uses true numerical information or if it relies on stimulus’s non-numerical physical features that covary with numerosity. Groups of objects with different numerosity usually differ for continuous proprieties, such as the cumulative surface area, the luminance, the objects’ density, and the array’s width. All the species studied thus far, including the human species, can solve a numerical task by relying on this continuous information or by combining continuous and numerical information to increase estimation accuracy^6,42^. Thus, proving the existence of true numerical abilities requires a series of careful control experiments in which access to non-numerical information is prevented^43,45,46^. These controls are usually complex to achieve simultaneously, especially when the numerical ratio discriminated by subjects is low, as in the case of our zebrafish larvae. Therefore, our experiments used a set of well-established separate control conditions^33,44,47^. Results showed that subjects selected the larger quantity regardless of whether the stimuli have been paired for total surface area, density, or the width of the array (as measured by its convex hull). While this result does not exclude that larvae can also process continuous quantities, it indicated that they used and prioritised numerical information in this context.

Among vertebrates, numerical abilities affect virtually all fitness-related functions (e.g., foraging and predation^48^; reproduction^49^; parental care^50^; antipredator defence^51^; inter-group conflicts^52^; habitat selection^3^) and not infrequently, they serve multiple functions within the same species^53–55^. In many of these examples, there appears to be a clear advantage in using numerical information rather than continuous physical attributes that covary with numerosity. For example, when gauging the social groups’ size^52,56^, luminance or cumulative surface are unreliable indicators of numerosity because retinal image size varies considerably for close and distant individuals and closer individuals may partly occlude those placed behind. Convex hull is also highly unreliable in this contest as it varies considerably with interindividual distance. Similar arguments can be made for foraging, habitat selection, or spatial navigation^33,54,57^.

In the aforementioned situations, pure numerical abilities provide reliable information for adaptive decisions. Therefore, it is not difficult to explain their presence in animals that are requested to perform such decisions, including adult and juvenile zebrafish^58,59^. What is puzzling, is the presence of numerical abilities in zebrafish at an early developmental stage when they seem to have reasonably no functional role. Larval zebrafish’s development is similar to that of altricial species in many respects. Hatching 3 days after fertilisation, newborn zebrafish are considered at a non-feeding eleutheroembryo stage, a post-hatching extension of embryonic development^60,61^. Their nervous system is relatively poorly developed at birth, to the point that the zebrafish display only simple responses to stimuli such as pain^62^. Other behaviours, such as feeding and efficient swimming, will appear after the sixth day post-fertilization and more complex behaviour such as social interactions occur only three weeks later^63–65^. Given their peculiar developmental mode, the most likely explanation for the presence of numerical abilities in zebrafish larvae is an innate biological predisposition. In line with the fitness advantages of processing numerical information^66^, such hardwired numerical abilities might prepare individual zebrafish for important behaviour but only in later life.

Our study’s conclusion is similar to that drawn for the presence of numerical abilities in human foetuses, when it is obviously not exploited^30^. That two distantly related vertebrates, a mammal and a teleost fish, show signatures of numerical abilities at a very early stage of their neural development supports the idea that numerical abilities might be hardwired in the vertebrate brain. In agreement with what has been proposed for other cognitive abilities^67–69^, the early development of numerical capacities might be essential to lay the foundation for more complex skills^70^, and therefore, it might be promoted by natural selection. Under this scenario, selection might have then determined the evolution of interspecific differences in numerical abilities by acting on the same, shared circuits for numerical processing or in additional circuits. Certainly, confirming this hypothesis will require more data on the larval zebrafish based on different stimuli (e.g., horizontal bars and other objects that do not resemble vegetation) and numerical tests exploiting other behaviours (e.g., foraging). Potentially, even more sophisticated, but less ecological, procedures might be adopted in this species, such as those used to test the recognition of absolute numerosity^71^. Extending the range of species investigated will be also paramount, with particular attention to those that permit associating behaviour with genetic and neurobiological data to infer homology of cognitive functions. In this light, the powerful research tools recently developed in zebrafish larvae (e.g., single-cell resolution functional brain imaging and cost-effective mutagenesis) also open a new window of opportunity.

## Methods

### Ethics

This research adheres to the ASAB/ABS Guidelines for the Use of Animals in Research^72^. The experiments were conducted in compliance with the Italian legislation (Italy, D.L. 4 Marzo 2014, n. 26) and were approved by the Ethical Committee of the University of Padova (Protocol No. 18/2018).

### Experimental subjects and rearing conditions

Subjects were zebrafish larvae with an age of 7 dpf (days post fertilisation). At this age, the visual system of zebrafish is almost completely developed and they swim constantly, thus allowing behavioural measurements^65^. We obtained the larvae collecting eggs from 14 breeding pairs. The adults used for breeding belonged to an outbreed laboratory population originated from individuals purchased at a local shop. These fish did not undergo genetic manipulation (wild-type). After collection, the eggs were kept in Petri dishes (Ø 10 cm, h 1.5 cm) filled with FishWater 1×^73^. Upon hatching (72 hpf), the larvae were moved to plexiglass aquaria (10.5 × 20.5 × 10 cm; Fig. 1a). Each rearing aquarium housed 50 larvae and was filled with 6 cm of FishWater, daily monitored to ensure the following parameters: temperature 29 ± 1 °C, nitrite levels < 0.1 mg/L, general hardness 5–10°d, pH 7.0, photoperiod 14/10D. The larvae were fed twice a day ad libitum with commercial food (Aqua Tropical, Isola Vicentina, Italy). Thirty minutes after feeding, the tank was cleaned with a Plastic pipette to remove food surplus, faecal material, and dead individuals. After 4 days under these rearing conditions, the experiments began (7-dpf). For the first experiment of this study, the rearing aquaria of half of the larvae had white walls. The remaining subjects used in the study were kept in rearing aquaria with white walls covered with a pattern of vertical black bars differing in size, width, and inter-bar distance (width range: 0.2–0.4 cm; height: 7 cm; inter-bar distance range: 0.1–1.8; Fig. 1a).

### Behavioural testing

We tested a total of 360 zebrafish larvae (see details below). The experimental apparatuses were eight identical plexiglass tanks (8 × 4 × 5 cm) filled with 90 mL of FishWater 1× and positioned into an empty white box (20 × 50 × 32 cm) to reduce external interference. Two LED strips (Eglo art.97572, 40.000 K, White) provided uniform illumination in each apparatus. We presented the stimuli in correspondence of the two short walls of apparatus (Fig. 1b), whereas the remaining part of the apparatus was covered with white paper preventing the fish from seeing the other tanks. The bottom of the apparatus was white to prevent scototaxis behaviour^74^. The stimuli (4 × 5 cm) were two-dimensional vertical black bars on a white background, draw using Microsoft PowerPoint and printed with a laser printer (Kyocera TASKalfa 4052ci).

At the beginning of the experiment, the subject was transferred into the centre of the experimental apparatus and left free to swim and interact with the stimuli. Testing time was 10 min in the first experiment, and 6 min in the following experiments, because data suggests that the latter time window was the most relevant for our analysis. A Canon LEGRIA HFR38 camera positioned 30 cm above the apparatus recorded the test since subject’s insertion.

We analysed subjects’ behavioural preference from the digital recordings played back on a computer. To assess spontaneous preference for the stimuli, we virtually divided the apparatus in three sectors; two 2 × 4 cm choice sectors facing the stimuli and one 4 × 4 cm central, no-choice sector (Fig. 1b). An experimenter blind with respect of the position of the stimuli analysed the video recordings at 2× speed using the computer software ‘Ciclic Timer’ (written in Delphi 5 Borland). The software allows to quantify the time spent in each choice sector of the tank.

### Bar stimuli

In the first experiment, we administered the choice between three bars on a white background (single size item 0.3 × 3 cm separate by 0.6 cm) and a white stimulus with no bars to test for a general preference for the bar stimuli. We tested 30 subjects from each treatment (experience with bars and control), for a total of 60 subjects.

To study numerical discriminations, we first used a set of three numerical discriminations with one bar as the smaller numerosity: 1 vs. 4, 1 vs. 3, and 1 vs. 2 bars. We used low numerical ratios (i.e., 0.25, 0.33, and 0.5) because larval subjects were expected to have reduced discrimination thresholds. We used small numerosities (1, 2, 3, and 4 bars) because the larvae have reduced mobilities and more stimuli would require larger apparatuses. All the bars were black and presented on a white background, and bars of the same stimulus were equally spaced. For half of the subjects, the stimuli were formed by bars of the same size (width 0.3 cm, height 3 cm). For these stimuli, the distance between the bars in the stimulus with the larger quantity was 0.6 cm (Fig. 3A). For the remaining half of subjects, the discriminations involved stimuli controlled for the cumulative surface area (Fig. 3A): the single bar in the stimulus with the smaller numerosity varied in surface area to match the cumulative surface area of the cluster of bars in the stimulus with the larger numerosity. In detail, the control for stimulus’s cumulative surface area was conducted as follow: in the 1 vs. 4 discrimination task, we presented four 0.3 × 3 cm bars separated by 0.6 cm (cumulative surface area: 3.6 cm^2^) and one 1.2 × 3 cm bar (cumulative surface area: 3.6 cm^2^); in the 1 vs. 3 discrimination task, we presented three 0.3 × 3 cm bars separated by 0.6 cm (cumulative surface area: 2.7 cm^2^) and one 0.9 × 3 cm bar (cumulative surface area: 2.7 cm^2^); in the 1 vs. 2 discrimination task, we presented two 0.3 × 3 cm bars separated by 0.6 cm (cumulative surface area: 1.8 cm^2^) and one 0.6 × 3 cm bar (cumulative surface area: 1.8 cm^2^). Using a photometer (Light Meter ST-1301, Resolution: 0.1 lux/Fc, Accuracy: ±5% of reading < 10.000 lux), we measured the luminance of the stimuli, finding that this variable significantly covaried with the cumulative surface area (rho = 0.97, *t*_7_ = 9.81, 95% CI [0.84, 0.99], *P* < 0.001). Therefore, half of our stimuli resulted also controlled for luminance (*t*_4_ = 0.82, 95% CI [−0.06, 0.11], *P* = 0.458). In this experiment, we tested 30 subjects for each condition (total: 180 subjects).

In the last set of numerical discriminations, we used four types of 2 vs. 4 stimuli, differing for the type of continuous variable controlled (Fig. 4A). To control for stimulus density, we used 0.3 × 3 cm bars separated by 0.6 cm in both the larger and the smaller stimulus. To control for cumulative surface area, we presented four 0.3 × 3 cm bars separated by 0.6 cm (cumulative surface area: 3.6 cm^2^) and two 0.6 × 3 cm bars separated by 0.6 cm (cumulative surface area: 3.6 cm^2^). To control for the convex hull, we presented four 0.3 × 3 cm bars separated by 0.6 cm and two four 0.3 × 3 cm bars separated by 2.4 cm. To simultaneously control for the cumulative surface area and the convex hull, we presented four 0.3 × 3 cm bars separated by 0.6 cm (cumulative surface area: 3.6 cm^2^) versus two 0.6 × 3 cm bars separated by 1.8 cm (cumulative surface area: 3.6 cm^2^). In this last experiment, we tested 30 subjects for each condition, for a total of 120 subjects.

### Statistics and reproducibility

The statistical analyses were performed in RStudio version 1.2.5019^75^. Descriptive statistics in the text are mean ± standard deviation. Statistical tests were two-tailed and *P* ≤ 0.05 was considered statistically significant. The dependent variable used to infer the preference between two stimuli was the time spent in each choice sector. This dependent variable was used to deal with substantial variability in subjects’ choosing time. To improve clarity, we also reported a percentage preference in the text calculated as: time spent close to the larger stimulus/(time spent close to the larger stimulus + time spent close to the smaller stimulus) × 100. Therefore, for each subject, the dependent variable had a repeated measure structure with two values (time spent in each choice sector). We analysed this dependent variable with linear mixed-effects models (‘lmer’ function from the ‘lmerTest’ R package^76^), which handle highly correlated multi-level data structure^77^ and reliably estimate parameters even in case of departures from distributional assumptions^78^. In all the models, subject ID was fitted as a random effect to account for the repeated measures. Stimulus (i.e., bars vs. empty or larger numerosity vs. smaller numerosity) was fitted as fixed effect; a significant effect of this term in the model would indicate a significant preference. Each model was also fitted with additional fixed effects of relevance such as the numerical discrimination (1 vs. 2, 1 vs. 3, 1 vs. 4) or the type of control (e.g., surface area, convex hull). We assessed the significance of the models’ parameters via Satterhwaite’s degrees of freedom method provided through the ‘anova’ function from the ‘lmerTest’ R package. Post-hoc paired t-tests were used to investigate significant interactions. In the first experiment of the study, we also analysed the temporal preference restricted to the subjects exposed to the vertical bars by using a model fitted with stimulus type and time blocks (i.e., 10 temporal blocks: ‘1’, ‘2’, …, ‘10’) as fixed effects, and subjects as random effect. In the second and third experiments, we also restricted the analyses to the first block of minutes. Inspection of residuals plots from the models used for analysing data of the first and third experiments violated the assumption of normality. Therefore, we square-root transformed the data and re-run the analyses. All sample sizes are reported in details in the description of each experiment, in Supplementary Information and in the figure captions.

### Reporting summary

Further information on research design is available in the Nature Portfolio Reporting Summary linked to this article.

## Supplementary information

Supplementary Information

Description of Additional Supplementary Files

Supplementary Data

Reporting summary

## Data Availability

The data of this study are available as Supplementary Data.
