# Supplementary material for: Quantity discrimination in newly hatched zebrafish suggests hardwired numerical abilities
Source: Commun Biol. 2023 Mar 23;6:247. doi: 10.1038/s42003-023-04595-7 (PMC10036331; doi:10.1038/s42003-023-04595-7)
Supplement: Supplementary file 1 — Supplementary Information [file 42003_2023_4595_MOESM1_ESM.pdf]

Quantity discrimination in newly hatched zebrafish suggests hardwired numerical abilities

Tyrone Lucon-Xiccato, Elia Gatto, Camilla Maria Fontana, Angelo Bisazza

Supplementary Information – Supplementary Tables

Supplementary Table 1.

Output of the linear mixed-effects model on time spent close to the stimulus with bars vs. the stimulus without bars (term 'Stimulus') with respect to subjects' rearing condition (aquaria with or without vertical bars; term 'Rearing condition').

Predictors	*Estimates*	*CI*	*P*
(Intercept)	8.85	7.52 – 10.18	**<0.001**
Rearing condition [Without bars]	-2.46	-4.34 – -0.58	**0.011**
Stimulus [Without bars]	-3.66	-5.54 – -1.78	**<0.001**
Rearing condition [Without bars] × Stimulus [Without bars]	5.11	2.45 – 7.76	**<0.001**
Random effects			
σ^2	13.49		
$\tau_{00\ subject}$	0.00		
$N_{subject}$	60		
Observations	120		

Supplementary Table 2.

Output of the linear mixed-effects model on the temporal variation (term ‘Block of time’) of time spent close to the stimulus with bars vs. the stimulus without bars (term ‘Stimulus’) by subjects reared in aquaria with bars.

Predictors	*Estimates*	*CI*	*P*
(Intercept)	4.48	3.70 – 5.26	**<0.001**
Stimulus [Without bars]	-3.42	-4.49 – -2.35	**<0.001**
Block of time	-0.28	-0.51 – -0.06	**0.015**
Stimulus [Without bars] × Block of time	0.54	0.22 – 0.87	**0.001**
Random effects			
σ^2	4.03		
$\tau_{00\ subject}$	0.29		
ICC	0.07		
$N_{subject}$	30		
Observations	300		

Supplementary Table 3.

Output of the linear mixed-effects models on the time spent close to the larger vs. smaller stimulus (term 'Stimulus') considering either the total testing time (left) or the first block of time (right). The model was also fitted with the stimuli's numerical ratio (term 'Ratio'; linear) and the type of continuous variable controlled for (term 'Control').

	**Total time**			**First block**		
Predictors	*Estimates*	*CI*	*P*	*Estimates*	*CI*	*P*
(Intercept)	53.99	48.68 – 59.30	**<0.001**	3.98	3.58 – 4.39	**<0.001**
Stimulus [Smaller quantity]	-14.13	-21.65 – -6.62	**<0.001**	-1.41	-1.99 – -0.84	**<0.001**
Ratio	-6.98	-16.18 – 2.22	0.137	-0.47	-1.17 – 0.23	0.189
Control [Cumulative surface area]	5.79	-1.72 – 13.30	0.131	0.33	-0.24 – 0.90	0.257
Stimulus [Smaller quantity] × Ratio	15.39	2.38 – 28.40	**0.021**	1.40	0.41 – 2.39	**0.006**
Stimulus [Smaller quantity] × Control [Cumulative surface area]	-21.59	-32.21 – -10.96	**<0.001**	-0.94	-1.75 – -0.13	**0.023**
Ratio × Control [Cumulative surface area]	-0.16	-13.18 – 12.85	0.980	-0.13	-1.11 – 0.86	0.803
Stimulus [Smaller quantity] × Ratio × Control [Cumulative surface area]	-5.42	-23.82 – 12.98	0.563	-0.18	-1.58 – 1.22	0.797
Random effects						
σ^2	656.52	3.79				
$\tau_{00\ subject}$	0.00	0.00				
$N_{subject}$	180	180				
Observations	360	360				

Supplementary Table 4.

Output of the linear mixed-effects models on the time spent close to the larger vs. smaller stimulus (term 'Stimulus') considering either the total testing time (left) or the first block of time (right). The model was also fitted with the type of continuous variable controlled for (term 'Control').

	Total time			First block		
Predictors	*Estimates*	*CI*	*P*	*Estimates*	*CI*	*P*
(Intercept)	7.11	6.35 – 7.86	**<0.001**	3.38	2.79 – 3.97	**<0.001**
Stimulus [Smaller quantity]	-1.50	-2.57 – -0.44	**0.006**	-0.53	-1.36 – 0.30	0.207
Control [Cumulative surface area]	-2.25	-3.32 – -1.18	**<0.001**	-0.91	-1.74 – -0.09	**0.031**
Control [Cumulative surface area and Convex hull]	-0.66	-1.73 – 0.41	0.223	0.29	-0.54 – 1.12	0.496
Control [Density]	-1.60	-2.66 – -0.53	**0.004**	-0.42	-1.25 – 0.41	0.320
Stimulus [Smaller quantity] × Control [Cumulative surface area]	-0.35	-1.86 – 1.16	0.649	-0.67	-1.84 – 0.51	0.264
Stimulus [Smaller quantity] × Control [Cumulative surface area and Convex hull]	0.16	-1.35 – 1.67	0.832	-0.97	-2.14 – 0.20	0.105
Stimulus [Smaller quantity] × Control [Density]	-0.46	-1.97 – 1.05	0.552	-0.87	-2.04 – 0.30	0.145

Random effects		
σ^2	4.40	2.66
$\tau_{00\ subject}$	0.00	0.00
$N_{subject}$	120	120
Observations	240	240
