# Supplementary material for: Quantity discrimination in newly hatched zebrafish suggests hardwired numerical abilities
Source: Commun Biol. 2023 Mar 23;6:247. doi: 10.1038/s42003-023-04595-7 (PMC10036331; doi:10.1038/s42003-023-04595-7)
Supplement: Supplementary file 2 — Description of Additional Supplementary Files [file 42003_2023_4595_MOESM2_ESM.pdf]

Description of Additional Supplementary Files

File Name: Supplementary Data

Description: The source data used for the analysis and the graphs in the paper
